# Gas-sensing behaviour of ZnO/diamond nanostructures

**DOI:** 10.3762/bjnano.9.4

**Published:** 2018-01-03

**Authors:** Marina Davydova, Alexandr Laposa, Jiri Smarhak, Alexander Kromka, Neda Neykova, Josef Nahlik, Jiri Kroutil, Jan Drahokoupil, Jan Voves

**Affiliations:** 1 Institute of Physics v.v.i., Academy of Sciences of the Czech Republic, Na Slovance 2, 18221 Prague, Czech Republic; 2 Department of Microelectronics, Faculty of Electrical Engineering, CTU in Prague, Technicka 2, 16627 Prague, Czech Republic; 3 Institute of Physics v.v.i., Academy of Sciences of the Czech Republic, Cukrovarnicka 10, 16200 Prague, Czech Republic

**Keywords:** density functional theory (DFT), gas sensor, interdigital electrodes, nanocrystalline diamond, sensitivity, zinc oxide (ZnO)

## Abstract

Microstructured single- and double-layered sensor devices based on p-type hydrogen-terminated nanocrystalline diamond (NCD) films and/or n-type ZnO nanorods (NRs) have been obtained via a facile microwave-plasma-enhanced chemical vapour deposition process or a hydrothermal growth procedure. The morphology and crystal structure of the synthesized materials was analysed with scanning electron microscopy, X-ray diffraction measurements and Raman spectroscopy. The gas sensing properties of the sensors based on i) NCD films, ii) ZnO nanorods, and iii) hybrid ZnO NRs/NCD structures were evaluated with respect to oxidizing (i.e., NO_2_, CO_2_) and reducing (i.e., NH_3_) gases at 150 °C. The hybrid ZnO NRs/NCD sensor showed a remarkably enhanced NO_2_ response compared to the ZnO NRs sensor. Further, inspired by this special hybrid structure, the simulation of interaction between the gas molecules (NO_2_ and CO_2_) and hybrid ZnO NRs/NCD sensor was studied using DFT calculations.

## Introduction

Currently, a number of studies have been focused on developing gas sensors based on nanomaterials and/or nanostructures. Metal oxides are the most common sensing materials and a variety of studies have been carried out to improve the response of these gas sensors [1–4]. In particular, ZnO nanorods with n-type semiconducting behaviour and large surface-to-volume ratio have attracted great interest due to their wide range of application possibilities in solar cell electrodes, light emitting devices, quantum dots and gas sensors [5–13]. The improvement in response of metal-oxide sensors has been observed by several authors, due to the formation of isolated functional layers on the one-dimensional nanostructure surface using metal oxides or noble metals [14–17]. Aside from n-type semiconductors, p-type semiconductor materials have also been extensively used for the detection of toxic gases [3,18–19]. Recently, nanocrystalline diamond (NCD) films have been utilized for advanced electronic devices because of their remarkable semiconducting properties [20–21]. For instance, hydrogen-terminated NCD films exhibit changes in their surface conductivity in the presence of phosgene and could be utilized as an integrator-type gas sensor [22–23]. Up to now, many research groups have focused on nitrogen dioxide (NO_2_) sensing. For instance, a hydrogen-terminated nanocone array exhibited a fast response time (4.7 s) towards 10 ppm of NO_2_ at 150 °C [13]. On the other hand, a room-temperature-operated gas sensor based on H-terminated diamond films showed a long response time and recovery time towards nitrogen dioxide [24]. Sadek et al. fabricated a ZnO nanobelt sensor and tested it for NO_2_ gas at operating temperatures between 150 and 450 °C. The optimum operating temperature for NO_2_ detection was in the range between 300 °C and 350 °C [25]. The sensing properties of various ZnO nanostructures (ZnO nanowires and ZnO–SnO_2_ core–shell nanowires) were investigated by Hwang and co-workers. The gas response of ZnO–SnO_2_ core–shell nanowires to 10 ppm NO_2_ at 200 °C and 300 °C were 66.3 and 12.4, respectively, which is ca. 33- and ca. 8.9-times higher than the respective values of 2.0 and 1.4 for ZnO nanowires [26].

Presently, materials with hybrid components have become more popular in the field of sensing because the hybridization and synergic effect of organic or inorganic materials could enhance the gas sensing properties. For example, Wang et al. fabricated a ring-like PdO–NiO composite with a lamellar structure and showed a rapid response speed (2 s) upon exposure to CO [27]. Zhang et al. synthesized hybrid ZnFe_2_O_4_/ZnO hollow spheres that exhibited a high sensitivity to acetone and a fast response speed of 5.2 s [28].

In this study we combine the merits of two different materials (n-type ZnO and p-type H-terminated diamond) in order to investigate their gas sensing properties for nitrogen dioxide (NO_2_), ammonia (NH_3_) and carbon dioxide (CO_2_) at different concentration ranges of 25–100 ppm or 1250–5000 ppm. For this purpose we developed three different gas sensor devices based on i) NCD thin films, ii) ZnO nanorods, and iii) a hybrid structure of ZnO nanorods combined with a diamond thin layer. The combination of ZnO nanorods with nanocrystalline diamond (hybrid ZnO NRs/NCD sensor) is analysed and discussed with respect to its gas sensitivity. Moreover, a simulation of the interaction between gas molecules and the hybrid ZnO NRs/NCD sensor is carried out using density functional theory (DFT) calculations.

## Experimental

**Figure 1:**
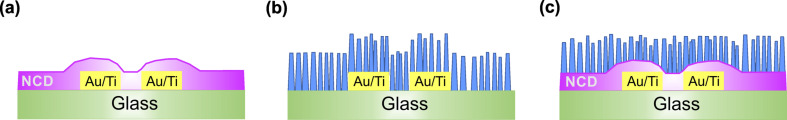
Schematic drawing of the sensor assembly designs: (a) continuous NCD film, (b) ZnO nanorods, and (c) ZnO nanorods combined with NCD thin layer.

Three different sensor designs were utilized with width and spacing of Au/Ti metal interdigital electrode (IDE) arrays of 100 μm. A schematic illustration of the sensor platforms is shown in Figure 1. All designs were fabricated in-house by standard UV lithography, followed by thermal evaporation and lift-off. The diamond growth was carried out by microwave-plasma-enhanced chemical vapour deposition from a gas mixture containing 1% of CH_4_ in H_2_ at temperatures as low as 450 °C. Then, the samples were exposed to pure hydrogen plasma for 10 min in order to create the p-type surface conductivity (Figure 1a) [29]. Vertically aligned 1-D ZnO nanorod arrays were synthesized either on a bare IDE glass substrate (Figure 1b) or on an IDE glass substrate covered with a diamond thin film (Figure 1c) by hydrothermal synthesis process. The synthesis was conducted in an equimolar aqueous solution containing zinc nitrate hexahydrate (Zn(NO_3_)_2_·6H_2_O) and hexamethylenetetramine (C_6_H_12_N_4_). During the synthesis a temperature of 90 °C was maintained for 3 h. The experimental procedure has been described in detail in [30].

The resulting morphologies of structured NCD films and ZnO NRs were characterized by field-emission scanning electron microscopy (FE-SEM, Merlin, ZEISS). Raman spectroscopy measurements were carried out at room temperature using a Renishaw InVia Raman Microscope with the following conditions: laser excitation wavelength of 488 nm (25 mW), 50× Olympus objective, 65 μm slits, spot focus, and grating of 2400 l/mm. The grazing-incidence XRD pattern was measured on a PANalytical diffractometer X`Pert PRO in parallel beam geometry by 2-theta scans with a fixed incident angle equal to 2.5°. The Co tube (λ = 0.178901 nm) and Göbel mirror in primary beam and parallel plate collimator with divergence 0.09° in diffracted beam were used.

The gas sensing experiments for all sensor designs were performed in an airtight chamber with electrical feedthroughs. The electrical resistance of the sensor devices was measured by source meter (Keithley 2400) in constant-current operation using a computer-controlled measurement system. A custom-written LabView program was used, which allowed temperatures and gas-flow rates to be automatically controlled by a computer. Prior to the measurements, the sensors were placed inside a constant-temperature chamber with a volume of 50 cm^3^ and flushed with dry nitrogen gas (N_2_) to stabilize the output characteristics. Next, the specific testing gas was injected into the chamber through the inlet port, and the change in the resistance of the sensors (∆*R*/*R*_0_) was investigated as a function of the exposure time. The sensor response is given by the relative resistance change, ∆*R*/*R*_0_ = (*R*_g_ − *R*_0_)/*R*_0_, where *R*_g_ and *R*_0_ are the resistances upon exposure to the specific gas and the reference gas (N_2_), respectively. The fabricated sensors were tested for carbon dioxide (CO_2_), nitrogen dioxide (NO_2_) and ammonia (NH_3_) at different concentrations at a temperature of 150 °C. The constant gas flow of 100 mL/min was maintained during all the measurements.

## Results and Discussion

### Materials characterization and gas sensing

**Figure 2:**
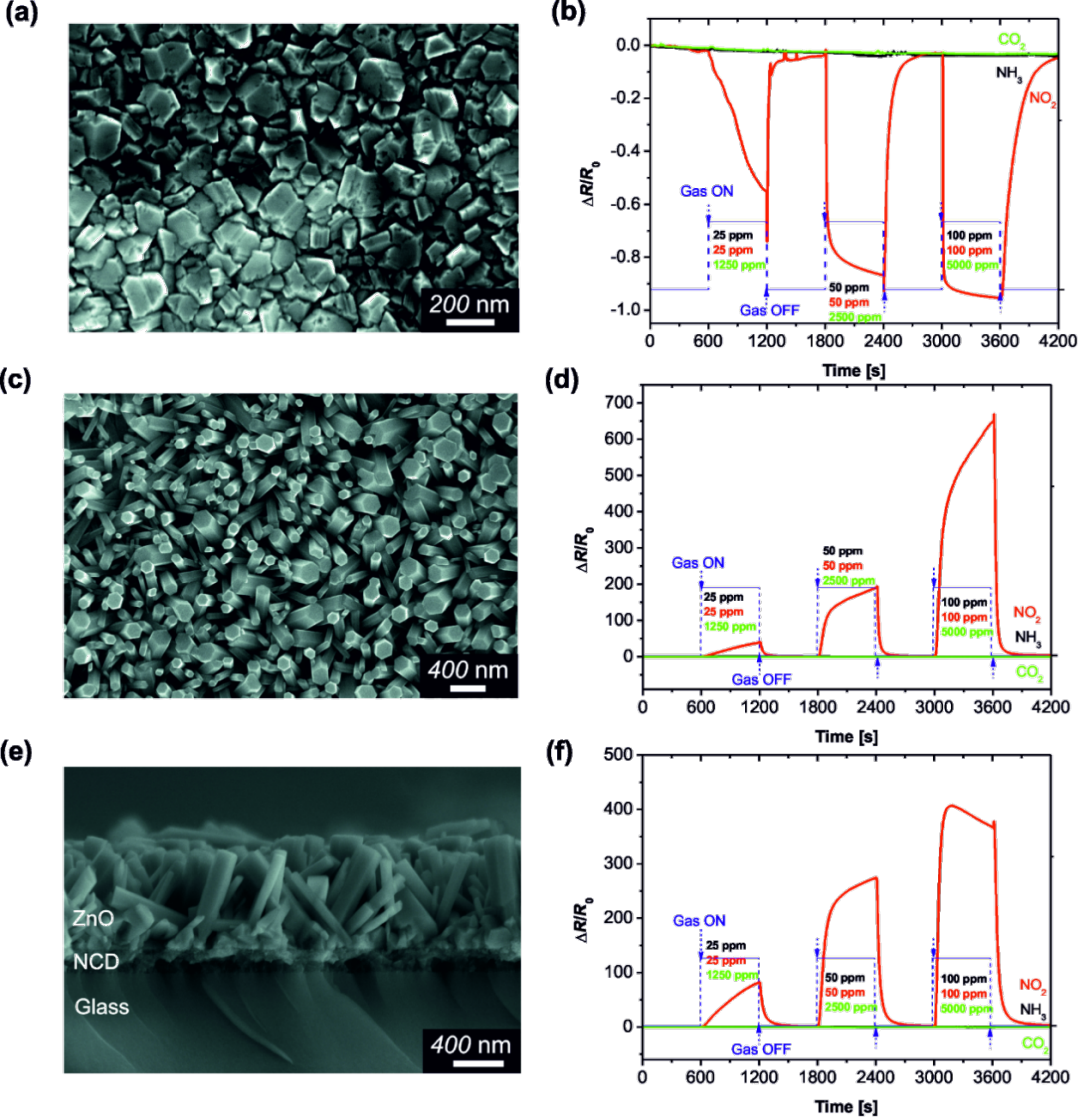
SEM surface morphology and corresponding plot of sensor response as a function of the time at a fixed temperature of 150 °C: (a,b) NCD thin film, (c,d) ZnO nanorods, (e,f) hybrid ZnO NRs/NCD-coated sensor substrate with Au/Ti IDE.

Figure 2a shows a top-view SEM surface morphology image of the nanocrystalline diamond layer deposited on the sensor substrate (glass + IDE). The mean grain size of the diamond crystals is about 100 nm. The responses of the p-type hydrogenated NCD sensor to CO_2_, NO_2_ and NH_3_ at various concentrations are shown in Figure 2b.

It can be seen that a decrease in the sensor resistance (sensor response) occurs in the presence of NO_2_, while negligible responses are detected during exposure to NH_3_ and CO_2_. In contrast to the NCD sensor, the sensor device based on ZnO nanorods acts as an n-type semiconductor [10]. Figure 2c shows a typical top-view SEM surface morphology image of the ZnO nanorods synthesized on the sensor substrate (glass + IDE). It was found that the hexagonal ZnO nanorods grow vertically with high density, 650 nm in length, and with an average diameter of about 150 nm.

The gas-sensing properties of the ZnO nanorods were tested for the various gases at different concentrations (Figure 2d). Again, it can be seen that the temporal response of the sensing device with ZnO nanorods increases at a higher concentration of NO_2_. Furthermore, the ZnO-based sensor exhibits responses of about 40, 190, and 650 at 25, 50 and 100 ppm NO_2_, respectively.

The hybrid sensor device combining two different materials (n-type ZnO and p-type H-terminated diamond film) is presented in Figure 2e. The SEM cross-sectional image of the hybrid ZnO NRs/NCD sensor reveals the clear interface between the NCD layer and the ZnO NRs (Figure 2e). The thickness of the diamond film is about 100 nm and the average length of the as-grown ZnO nanorods is approximately 650 nm. The dynamic response–recovery curves of the hybrid ZnO NRs/NCD sensor to different gases are shown in Figure 2f. They indicate a reproducible and reversible sensing of NO_2_.

The experimental data lead to the conclusion that the sensor based on the hybrid ZnO NRs/NCD film shows not only quick response and recovery to NO_2_, but also saturation behaviour for concentrations above 50 ppm. The response of the sensor increased swiftly when exposed to the target gas (NO_2_) and recovered its initial value after the tested gas was removed. The responses towards 25, 50 and 100 ppm nitrogen dioxide are about 82, 274 and 406, respectively. Compared with the sensor device based on ZnO NRs, the hybrid ZnO NRs/NCD sensor has a higher sensor response upon exposure 25–50 ppm NO_2_ (Figure 2d and Figure 2f). Importantly, when the concentration of NO_2_ is higher than 50 ppm the sensor becomes saturated (Figure 2f) in comparison to the response of ZnO-based sensor (Figure 2d). It should be also noted that the negligible response to NH_3_ in N_2_ atmosphere (approx. 10% resistance decrease) can be explained by: i) slow reduction of a limited amount of adsorbed oxygen on the sensor surface, and ii) relatively low working temperature of 150 °C.

For the purpose of understanding the decrease or increase in resistivity of our sensor devices, the gas sensing mechanism has to be discussed. After exposing the sensors to oxidizing gas (i.e., NO_2_), the electric resistance decreases for p-type H-terminated NCD (Figure 2b); on the contrary, with n-type ZnO the resistance increases (Figure 2d). In general, this response behaviour is in concordance with the typical gas sensing mechanism of p- and n-type semiconductors [22,31]. The gas sensing properties of the hybrid sensor structure (Figure 2f) were further enhanced through the combination of nanocrystalline diamond and ZnO NRs, the conduction mechanism of which becomes rather complex. We assume that at the ZnO/NCD interface the electrons in the ZnO conduction band are attracted to the interface by the p-type NCD. Therefore, conductivity of this ZnO layer increases (see Figure S3 in Supporting Information File 1). Hence, the relative change of the resistivity of the hybrid ZnO NRs/NCD sensor is larger than that of the pure ZnO sensor. Simultaneously, the active area located at the ZnO/NCD interface is smaller than in the ZnO sensor. This could lead to a better sensitivity of the hybrid ZnO NRs/NCD sensor and a faster saturation.

To confirm the sensor sensitivity to NO_2_ the response to different concentrations of oxygen by mixing synthetic air with nitrogen was tested at 150 °C. The sensor responses (*∆R*/*R*_0_) towards 5%, 10% and 20% O_2_ are about 2.5, 5 and 10, respectively for the hybrid ZnO NRs/NCD sensor. This is a much smaller response than for NO_2_ (Figure 2f). Similar results were observed in [32].

**Figure 3:**
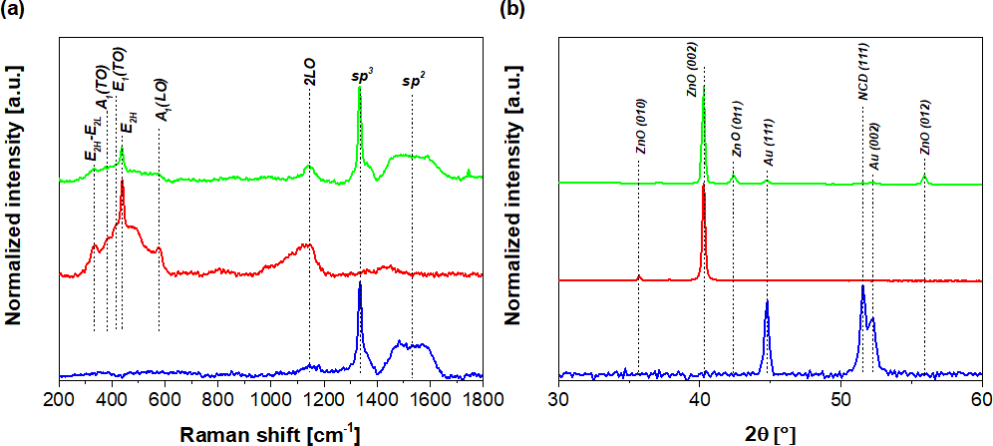
(a) Raman spectra and (b) X-ray diffraction patterns of sensor devices based on NCD thin film (blue line), ZnO NRs (red line), and hybrid structure ZnO NRs/NCD (green line).

Figure 3a and Figure 3b show the Raman spectra and X-ray diffraction (XRD) results of diamond thin layer, ZnO nanorods and hybrid ZnO NRs/NCD grown on the sensor substrate with metal interdigital electrodes. Figure 3a shows room-temperature Raman scattering spectra of the structures with ZnO nanorods and/or NCD under an excitation wavelength of 488 nm. The Raman spectrum of ZnO NRs is characterized by several peaks. Here, the visible modes peaked at 330, 378, 415, 437, 570 and 1130 cm^−1^ are assigned to the modes E_2H_-E_2L_, A_1_(TO), E_1_(TO), E_2H_, A_1_(LO) and 2LO, respectively [33–34]. The peak at 437 cm^−1^ is attributed to the E_2H_ mode, which is the intrinsic characteristic of the Raman active mode of wurtzite hexagonal ZnO. The Raman spectrum of diamond (Figure 3a) is characterized by two strong contributions. The peak centred at 1330 cm^−1^ corresponds to the diamond (sp^3^ hybridisation) component. The broad band at approximately 1580 cm^−1^ is attributed to the non-diamond phase (G-band), i.e., sp^2^-hybridised carbon atoms [35]. The high intensity of the diamond peak with respect to the G-band indicates a strong predominance of the diamond phase in the film with respect to the graphite component.

In the XRD diffractograms (Figure 3b), the main diffraction peaks can be indexed as hexagonal wurtzite ZnO with the lattice parameters *a* = 3.252 Å and *c* = 5.208 Å. On the other hand, the intensity of the (002) peak is the strongest; the other ZnO diffraction peaks exhibit a gradual weakening. Furthermore, the peak presented at 51.5° corresponds to the (111) crystal plane of diamond. In addition, the peaks presented at 44.8° and 52.3° are assigned to the (111) and (002) diffraction peaks of Au in the interdigitated electrodes.

### Simulation of interaction between gas molecules and hybrid ZnO NRs/NCD sensor structure

In order to better understand the gas sensor measurements, ab initio simulations of the interaction between NO_2_ or CO_2_ molecules and the hybrid ZnO NRs/NCD sensor were carried out doing first principle DFT calculations with the QuantumWise Atomistix ToolKit simulation package [36–38]. The DFT calculations were performed using the local density approximation (LDA) with the Perdew–Zunger parameterization of the correlation energy [39].

**Figure 4:**
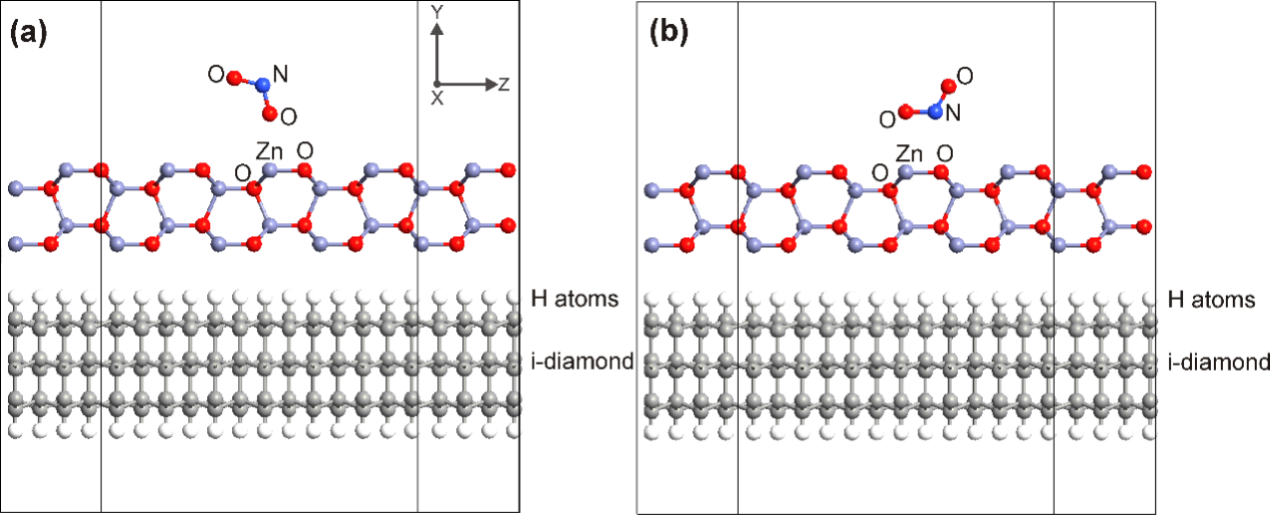
Simulated interaction between an NO_2_ gas molecule and the non-polar hybrid ZnO NRs/NCD sensor with two different orientations of the NO_2_ molecule: (a) T-shaped and (b) L-shaped.

The real structure consists of nanocrystalline layers with a thickness of hundreds of nanometers (Figure 4). In order to reduce atomic-scale simulation computational time, the simplifications had to be realized by: i) taking into account only the thin layer near the ZnO/diamond interface, ii) reducing the unit cell to the minimal size, and iii) choosing one of the probable gas molecule orientations.

The simulated device is composed of two electrodes and a central region. Since the method uses the border parts of the bulk to define the electrode regions, only one unit cell of ZnO is considered as the central region. For defining the electrode regions, the NCD is also reduced from both sides to make two and a half unit cells as a resulting central region.

Two different orientations of NO_2_ molecule above the ZnO surface were studied. First, the NO_2_ molecule is rotated to have one oxygen atom face the zinc atom of the surface (T-shape). In the L-shape the one oxygen atom of the gas molecule is also facing the zinc atom of the surface, but with the nitrogen atom parallelly oriented to the surface. The simulated structures consist of a 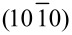-oriented ZnO layer and an intrinsic diamond (i-diamond) passivated by hydrogen on the 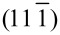 surface (Figure 4). The crystallographic orientation of the model ZnO surface was set to the real orientation of the nanorod sidewalls to better assess the influence of gas molecule interaction with the real hybrid ZnO NRs/NCD sensor structure.

Due to the used boundary conditions in the direction of the *Y*-axis, the lower side of diamond layer was also passivated by H atoms. We assume that the ZnO layer in the used orientation does not need to be treated by other atoms due to the boundary conditions in the directions of *X*- and *Z*-axis and the electrostatic stability of the ZnO 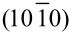 surface. Moreover, it should be pointed out that the structure was simulated without molecular mechanical calculations. It was found that the NO_2_ molecule is non-covalently bonded to the metal oxide surface atoms and van der Waals forces occur between gas molecule and ZnO surface [40]. Therefore, the distance between NO_2_ molecule and ZnO layer is set according to the van der Waals radius of the respective atoms. The same approach was applied to the ZnO/diamond interlayer distance. The double-zeta polarized basis set was used for all involved elements. The *k-*point sampling for DFT calculations was set to 2 × 2 × 20 to save computational time. The Poisson solver was set to direct method with the Dirichlet boundary conditions in directions *X* and *Z* and periodic boundary conditions in *Y*-direction.

The electron transport calculations were performed using DFT with non-equilibrium Green´s function formalism. The transmission function *T*(*E*, *V*) is a sum of the transmission probabilities of all channels available at energy *E* and it also depends on bias voltage *V*. We can apply this function to find the electric current by the Landauer–Buttiker formula [41]:

[1]
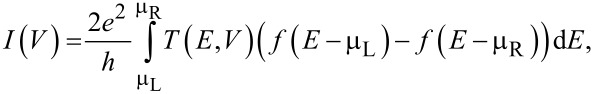


where *h* is Planck’s constant, *e* is the elementary charge, and μ_L_ and μ_R_ are the chemical potentials of the left and the right electrode, respectively. Therefore, the transmission in the small energy interval between the electrode chemical potentials determines the conductivity of the structure for low voltages applied. At room temperature or above the Fermi–Dirac distribution function *f* has non-zero values in a small energy interval above the Fermi level (approx. 0.1 eV for 25 °C and approx. 0.15 eV for 150 °C).

**Figure 5:**
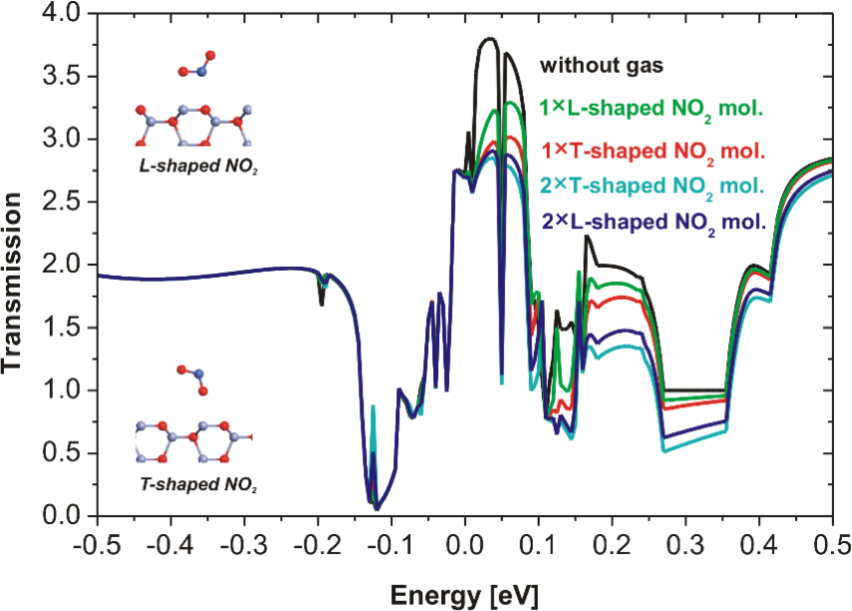
Transmission spectra of the hybrid ZnO/NCD structure without and with one or two NO_2_ molecules at two different orientation. Zero energy is the Fermi level. Transmission values determining the conductivity (resistivity) measured at 150 °C are within the range of 0–0.15 eV.

Figure 5 shows the dependence of the simulated transmissions on the energy of the hybrid ZnO/NCD sensor. It should be noted that the simulation was performed with different numbers of NO_2_ molecules (one or two) as well as various molecule orientations (L-shaped or T-shaped). It is evident that transmissions in the vicinity of the Fermi level are more sensitive to the T-shaped NO_2_ molecule than to the L-shaped molecule. The transmission decreases with increasing number of NO_2_ molecules.

**Figure 6:**
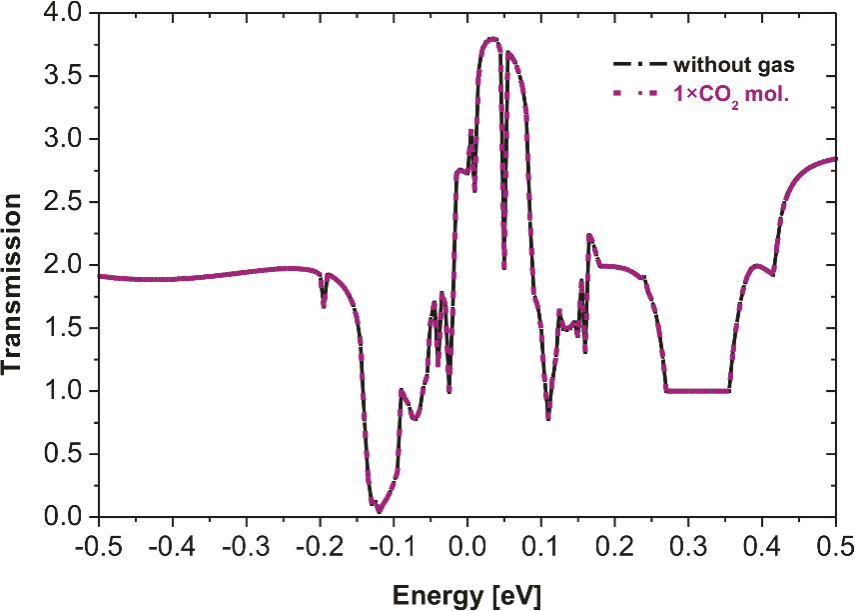
Transmission spectra of hybrid ZnO NRs/NCD sensor structure with and without one CO_2_ molecule. Zero energy is the Fermi level.

The simulated transmission with CO_2_ gas shows no change with respect to the results in the absence of gas (Figure 6). These results are in agreement with our gas sensing measurements where no response was found in the presence of CO_2_ (Figure 2b,d). All sensor types showed almost no response to NH_3_. Therefore, the time consuming atomistic simulations were omitted for this gas.

**Figure 7:**
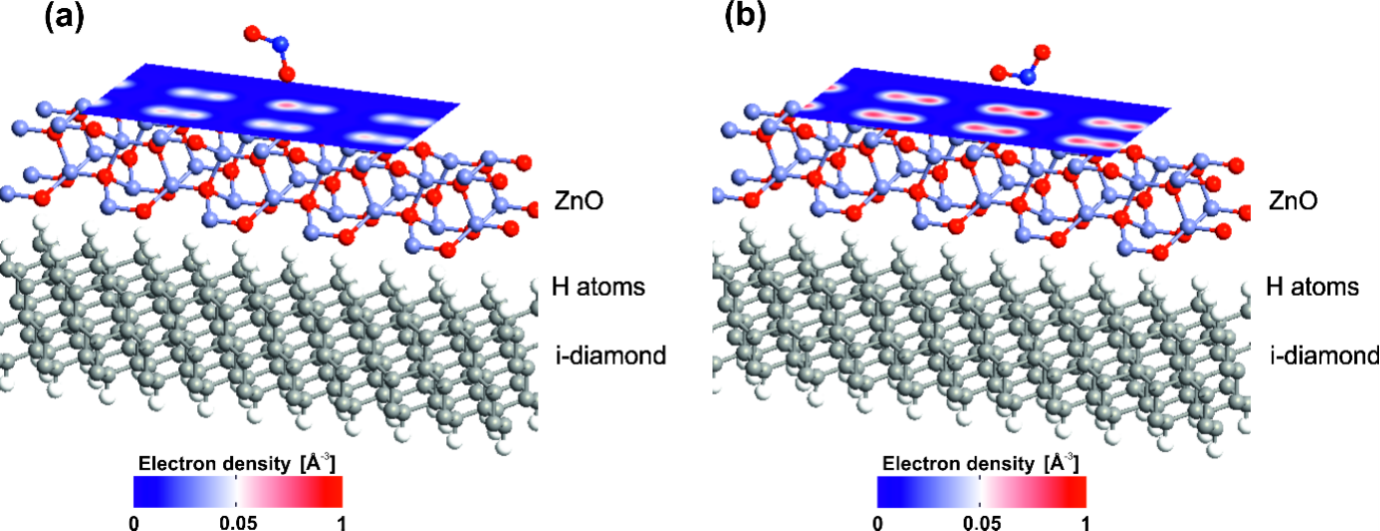
Electron density distribution of (a) T-shaped NO_2_ and (b) L-shaped NO_2_ molecules.

To support the above statements, we show the electron density horizontal cut for both NO_2_ molecule configurations (Figure 7). In the case of T-shaped NO_2_ molecule (Figure 7a) the simulated electron density is lower than that for L-shaped NO_2_ (Figure 7b), which gives rise to its higher resistivity.

According to the above results, all simulated transmissions by the simplified model show changes that are qualitatively consistent with the experimental results of the NO_2_ interaction with the hybrid ZnO NRs/NCD system. It was also found that the transmission decreases in the vicinity of the Fermi level of the system when the number of NO_2_ molecules is increased. It corresponds with the resistance increase at higher NO_2_ concentrations. The different shapes (T and L) of NO_2_ molecule have negligible influence on the transmission.

## Conclusion

In the presented work we reported on the fabrication of single- and double-layered sensor devices using p-type H-terminated nanocrystalline diamond film and n-type ZnO nanorods. It was observed that all sensor devices were sensitive to nitrogen dioxide. Moreover, the hybrid ZnO NRs/NCD sensor configuration remarkably enhanced NO_2_ gas sensor responses compared to the ZnO single-layer sensor (i.e., *∆R*/*R*_0ZnO/NCD_ = 82 at 25 ppm and *∆R*/*R*_0ZnO/NCD_ = 274 at 50 ppm, *∆R*/*R*_0ZnO_ = 40 at 25 ppm and *∆R*/*R*_0ZnO_ = 190 at 50 ppm). Furthermore, the experimental results of the interaction of NO_2_ and CO_2_ with the hybrid ZnO NRs/NCD sensor system were confirmed by DFT calculations. It was observed that transmission decreases in the vicinity of the Fermi level of the system when the NO_2_ concentration is increased, whereas for CO_2_ no change of transmission occurred. These preliminary results open new perspectives in the development of highly sensitive gas sensors based on hybrid nanostructures.

## Supporting Information

File 1 Additional experimental data.
