# Supplementary material for: Gas-sensing behaviour of ZnO/diamond nanostructures
Source: Beilstein J Nanotechnol. 2018 Jan 3;9:22–9. doi: 10.3762/bjnano.9.4 (PMC5769078; doi:10.3762/bjnano.9.4)
Supplement: File 1 — Additional experimental data. [file Beilstein_J_Nanotechnol-09-22-s001.pdf]

Supporting Information

for

Gas-sensing behaviour of ZnO/diamond nanostructures

Marina Davydova*[1], Alexandr Laposa[2], Jiri Smarhak[2], Alexander Kromka[3], Neda Neykova[3], Josef Nahlik[2], Jiri Kroutil[2], Jan Drahokoupil[1] and Jan Voves[2]

Address: [1] Institute of Physics v.v.i., Academy of Sciences of the Czech Republic, Na Slovance 2, 18221 Prague, Czech Republic; [2]Department of Microelectronics, Faculty of Electrical Engineering, CTU in Prague, Technicka 2, 16627 Prague, Czech Republic and [3]Institute of Physics v.v.i., Academy of Sciences of the Czech Republic, Cukrovarnicka 10, 16200 Prague, Czech Republic

Email: Marina Davydova* - davydova@fzu.cz

* Corresponding author

Additional experimental data

The schematic diagram of the experimental gas sensing setup is shown in Figure S1. The test procedure consists of two fundamental phases: the purging and the detection phase. During the first phase (purging), a pure carrier gas flows through the test chamber to obtain a baseline. The stable level of pure carrier gas flow was controlled by a Bronkhorst programmable mass flow controller (MFC #1). The concentration of tested gas was controlled by mixing the ratio of tested gas on MFC #3 and pure carrier gas on MFC #2. The gas flows were kept at the level of 100 ml/min in both purging and detection phases. Switching between the purging and detection phase was done by 2-way 4-port VICI valve. Volume of the test chamber is approximately 50 cm3. The commercial TELPOD ceramic heating element was used which allows it to reach a temperature of up to 500 °C.

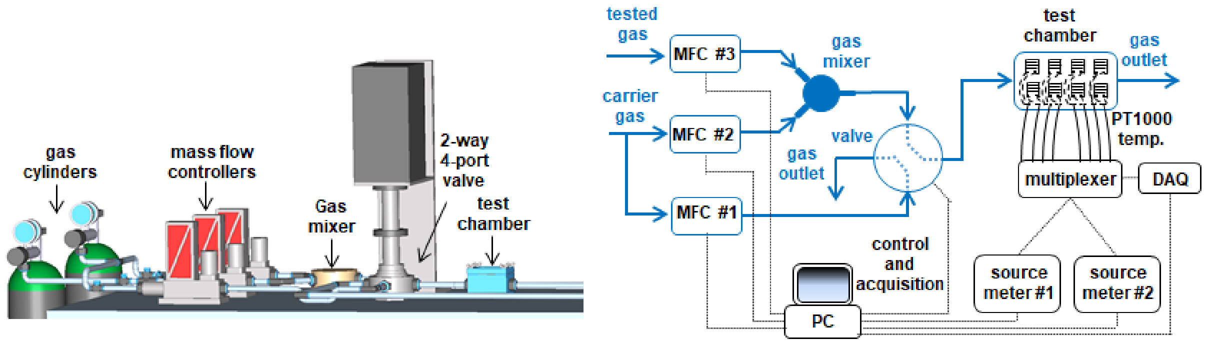

Figure S1: Schematic diagram of the gas sensing setup.

Figure S2 shows the response and recovery time of hydrogen terminated NCD, ZnO NRs and hybrid ZnO NRs/NCD sensors for different concentrations of NO_2 at 150 °C. It is evident that with increasing NO_2 concentration (25-100 ppm) the response time decreases from 482 to 20 s and from 493 to 62 s for hydrogen terminated NCD and hybrid ZnO NRs/NCD sensors, respectively. However, the response time of ZnO NRs

sensor slows down when NO_2 concentration increases from 25 to 50 ppm, and reach saturation with further increasing of gas concentration.

The recovery time of NCD sensor becomes 3.5 times longer as the gas concentration increased from 25 to 100 ppm. Whereas, a significant decrease in the recovery time with increasing gas concentration have been found for ZnO NRs and hybrid ZnO NRs/NCD sensors (Table S1).

Table S1: The response/ recovery time towards different concentration NO_2.

Gas concentration [ppm]	Response time [s]			Recovery time [s]		
	NCD	ZnO NRs	ZnO NRs/NCD	NCD	ZnO NRs	ZnO NRs/NCD
**25**	482	530	493	82	134	145
**50**	107	406	269	141	58	92
**100**	20	412	62	306	50	111

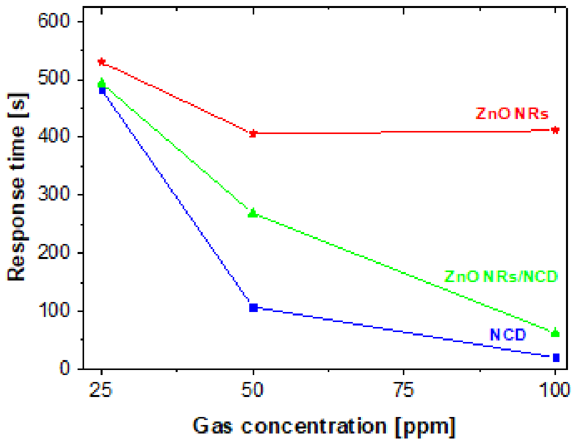

(a)

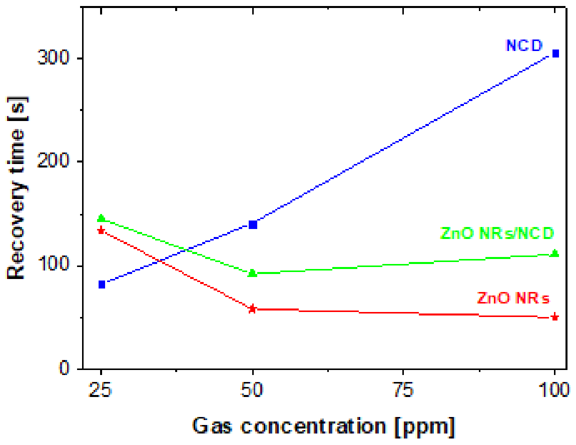

(b)

Figure S2: Response (a) and recovery (b) time as a function of the NO_2 gas concentrations for all sensors.

The model presented in Figure S3 is based on Silvaco Atlas device simulator with following parameters: hydrogenated diamond electron affinity = -1.3eV, ZnO affinity = 4.6 eV, ZnO effective donor concentration = 10^{15} cm^{-3}, NCD surface layer effective

p=type concentration = 10^{14} cm^{-3}. The NO_2 exposition is simulated by the floating gate above the ZnO surface with negative charge. The change in the electron concentration after NO_2 exposition is larger for the hybrid ZnO NRs/NSD sensor structure than for the pure ZnO case (graph is in the log scale). Sensor conductivity is proportional to the integral of electron concentration across the active area.

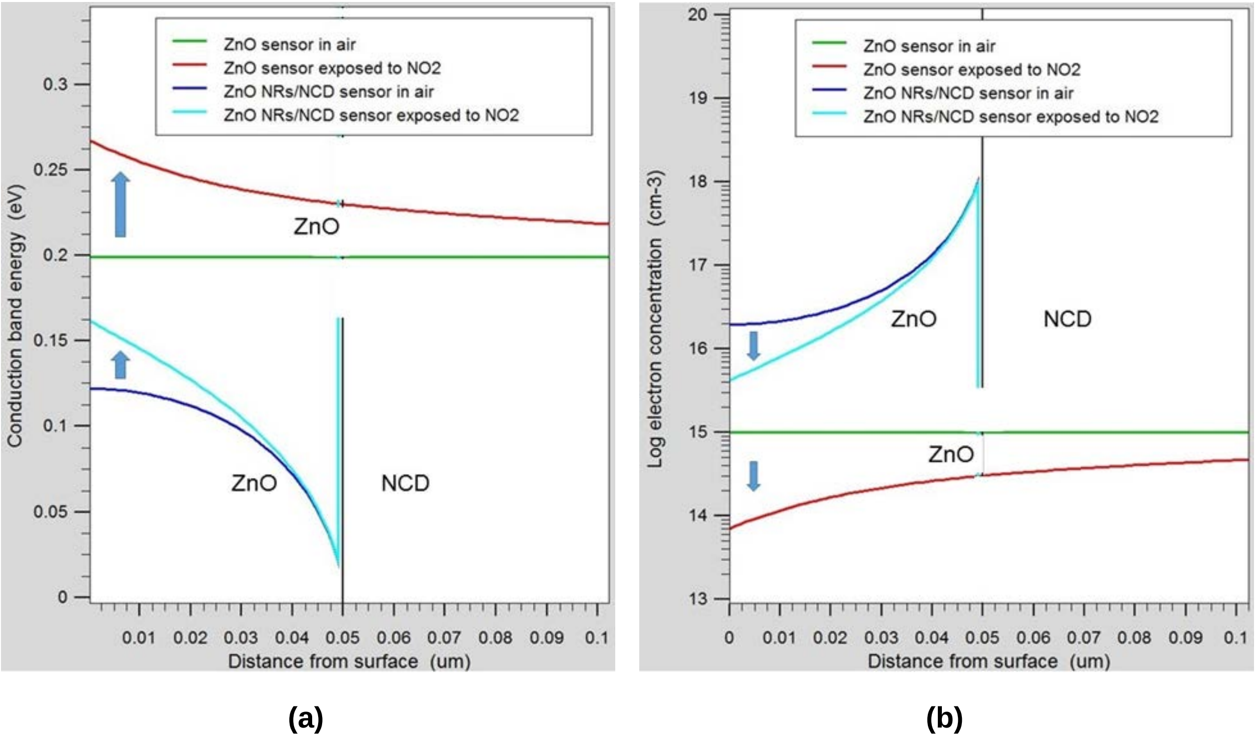

Figure S3: (a) Conduction band energy and (b) logarithm of electron concentration calculated by simple model of ZnO sensor and hybrid ZnO NRs/NCD sensor structures in air and by exposition to NO_2 gas.
